# Massage Therapy Modulates Inflammatory Mediators Following Sprint Exercise in Healthy Male Athletes

**DOI:** 10.3390/jfmk5010009

**Published:** 2020-01-29

**Authors:** Gillian E. White, Sarah L. West, Jessica E. Caterini, Alex P. Di Battista, Shawn G. Rhind, Greg D. Wells

**Affiliations:** 1 Graduate Department of Exercise Sciences, University of Toronto, Toronto, ON M5S 2W6, Canada; Jessica.caterini@sickkids.ca (J.E.C.); Alex.di.battista@utoronto.ca (A.P.D.B.); Shawn.Rhind@drdc-rddc.gc.ca (S.G.R.); 2 Translational Medicine, The Hospital for Sick Children, Toronto, ON M5G 1X8, Canada; sarahwest@trentu.ca (S.L.W.); Greg.wells@sickkids.ca (G.D.W.); 3 Department of Biology & Trent/Fleming School of Nursing, Trent University, Peterborough, ON K9L 0G2, Canada; 4 Defence Research and Development Canada, Toronto Research Centre, Toronto, ON M3K 2C9, Canada

**Keywords:** massage therapy, recovery, inflammation, exercise

## Abstract

Massage therapy is a common postexercise muscle recovery modality; however, its mechanisms of efficacy are uncertain. We evaluated the effects of massage on systemic inflammatory responses to exercise and postexercise muscle performance and soreness. In this crossover study, nine healthy male athletes completed a high-intensity intermittent sprint protocol, followed by massage therapy or control condition. Inflammatory markers were assessed pre-exercise; postexercise; and at 1, 2, and 24 h postexercise. Muscle performance was measured by squat and drop jump, and muscle soreness on a Likert scale. Significant time effects were observed for monocyte chemoattractant protein-1 (MCP-1), interleukin-8 (IL-8), interleukin-6 (IL-6), interleukin-10 (IL-10), tumor necrosis factor alpha (TNFα), drop jump performance, squat jump performance, and soreness. No significant effects for condition were observed. However, compared with control, inflammatory marker concentrations (IL-8, TNFα, and MCP-1) returned to baseline levels earlier following the massage therapy condition (*p* < 0.05 for all). IL-6 returned to baseline levels earlier following the control versus massage therapy condition (*p* < 0.05). No differences were observed for performance or soreness variables. MCP-1 area under the curve (AUC) was negatively associated with squat and drop jump performance, while IL-10 AUC was positively associated with drop jump performance (*p* < 0.05 for all). In conclusion, massage therapy promotes resolution of systemic inflammatory signaling following exercise but does not appear to improve performance or soreness measurements.

## 1. Introduction

High-intensity exercise results in metabolic and mechanical stress to muscle fibers, leading to delayed-onset muscle soreness (DOMS) and/or increased perceived fatigue, which can be associated with performance decrements. These negative consequences of high-intensity exercise are thought to result from disruption of the muscle microstructure and subsequent inflammatory responses [1,2,3,4]. While the inflammatory response to intense exercise is important for recovery and repair of stressed or damaged muscle tissue [5], it is a positive feedback process, whereby stress signals in the form of inflammatory mediators are released from damaged or dysregulated muscle tissue. These activate inflammatory effector cells, ultimately leading to the release of systemic inflammatory signaling mediators—cytokines [5,6,7]. Excessive or prolonged inflammation can lead to edema, local hypoxia, and accumulation of noxious substances, as well as phagocytosis of healthy tissue by inflammatory cells, resulting in soreness and tissue disruption [6,8]. Therefore, many postexercise modalities have been used to facilitate quicker and better recovery postexercise by targeting inflammatory processes [9].

Massage therapy is one postexercise recovery technique that is widely used. Previous studies investigating the effects of postexercise massage on DOMS have reported consistent improvements in DOMS at 24 and 48 h postexercise [2,10]. As well, a recent meta-analysis revealed that massage therapy was the most effective technique in reducing perceived fatigue. Massage therapy is hypothesized to act by blunting the production of inflammatory signaling at the cellular level [8,11,12] while also promoting the clearance of inflammatory signaling factors such as cytokines from systemic circulation [13,14,15,16]. For example, Crane, et al. [12] used various massage techniques, including effleurage, petrissage, and slow stripping, following an interval training protocol in inactive participants. They found reduced expression of proinflammatory pathways (i.e., NFκB) and increased expression of anti-inflammatory and regenerative pathways (i.e., ERK1/2 and FAK) at 2.5 h after massage therapy. Animal studies have found that massage therapy can promote the shift in local inflammatory effector cell populations, such as macrophages, toward a phenotype promoting repair and regeneration of damaged muscle fibers [11]. These animal studies have also shown histological recovery of normal myofibrillar organization and recovery of function following massage [11], which suggest that manual manipulation of skeletal muscle by massage therapy can promote repair of muscle fibers and recovery of both structure and function.

However, the overall impact of massage on muscle recovery remains unclear, likely due to the variability in massage therapy protocols used [17,18]. Additionally, in studies that examine the impact of massage postexercise, the inflammatory processes are not commonly evaluated in tandem with physical performance and soreness metrics. Therefore, in the present study, we sought to understand the impact of massage therapy using effleurage and neurolymphatic activation techniques on postexercise inflammatory signaling and subsequent muscle performance and soreness resulting from an intense bout of high-intensity sprint exercise. Given the proposed mitigation of postexercise stress signaling and enhanced clearance of inflammatory signaling factors such as cytokines associated with massage therapy, we hypothesized that postexercise effleurage and neurolymphatic massage would reduce the time-course and area under the curve (AUC) of inflammatory signaling factors. Further, we hypothesized that lower inflammatory signaling would be associated with reduced DOMS and would blunt muscle performance decrements experienced following intense intermittent sprint exercise.

## 2. Materials and Methods

Male collegiate-level or comparable athletes volunteered to participate in the study (Table 1). Participants were not taking medications, did not report a history of anti-inflammatory use, were free of cardiopulmonary and/or inflammatory conditions, and were cleared by their athletic trainer to participate. All participants were in the off-season of their sport’s competitive cycle. The study was approved by the University of Toronto Research Ethics Board (Protocol Reference number 30423). Each participant completed a PAR-Q screening form (http://www.csep.ca/CMFiles/GAQ_CSEPPATHReadinessForm_2pages.pdf) and provided written consent to participate.

### 2.1. Experimental Overview

Participants completed a high-intensity intermittent sprint exercise protocol followed by either a massage therapy treatment or a control condition. Participants completed both the massage therapy and control conditions (on two different trial days, separated by 1 week), and the order of trial days was randomized and counterbalanced. Moderate- to high-intensity exercise (>12 on the Borg 20-point scale), alcohol, and caffeine were prohibited 24 h prior to any blood collection. Nonsteroidal anti-inflammatory drugs, steroidal anti-inflammatory drugs, and antioxidant supplementation were prohibited for the duration of the study. Anthropometric measures, maximal aerobic power (VO_2peak_), Wingate performance, and familiarization with the exercise protocol were conducted 1 week prior to the experimental (massage therapy or control) trial days. Drop jump and squat jump height, rating of perceived muscle soreness, and a blood sample were taken pre-exercise; postexercise; and at 1, 2, and 24 h postexercise. All test procedures were completed at the University of Toronto Athletic Centre. No food was consumed during the experimental procedures. Water was permitted ad libitum.

### 2.2. Exercise Protocol

The exercise protocol used in the current study has been shown to increase inflammatory signaling and has been described previously [19]. Briefly, the exercise protocol was conducted on a 200 m indoor track between the hours of 12:00 and 6:00 p.m. Participants attended sessions at the same time of day for each of the experimental trials (massage or control). Following pre-exercise measurements, participants completed a standardized warm-up including 800 m at a self-selected jogging pace, a series of eight dynamic stretches performed over 20 m, and a 5 min free-stretch period followed by the intermittent sprint protocol. The intermittent sprint protocol was comprised of 10 repetitions of 120 m distance sprints at maximal effort. Upon completion of the sprint, the participant was instructed to walk back to the start line on the opposing side of the track. Each sprint was initiated 3 min from the start of the previous sprint, such that the duration of rest between sprints equated to 3 min minus the time taken to complete the 120 m sprint (i.e., a 20 s sprint completion would allow 2 min and 40 s of rest).

This protocol was chosen to induce high metabolic and mechanical stress by engaging a large muscle mass with ground-contact forces and repeated stretch-shortening cycles. The rest period was chosen to allow full-recovery of phosphocreatine stores for maximal effort on subsequent repetitions. Heart rate and Rating of Perceived Exertion (RPE) were recorded at the beginning and end of each sprint to ensure maximal effort and to compare effort between trials. Standardized encouragement was given before each bout, with a reminder to exert maximal effort. Upon completion of the 10 sprint repetitions, participants completed a cool-down of 400 m jogging at a self-selected pace prior to returning to the laboratory for postexercise measurements and their assigned recovery treatment (massage or control) for that trial.

### 2.3. Massage Therapy Protocol

Immediately following postexercise blood sampling, performance, and soreness measurements, participants assigned to the massage therapy condition underwent the massage therapy protocol. The protocol was 30 min in duration and included traditional effleurage of the lower body muscles and the lower back muscles for 6 min, neurolymphatic activation for 18 min, followed by a further 6 min of effleurage of the lower body muscles. Treatments were administered by one of three Certified Sport Massage Therapists trained in the neurolymphatic activation technique. Neurolymphatic reflex points located along the spine, inner sternum, and outer spine and were activated using 0.5–1.5 kg force administered with the Lafayette Manual Muscle Testing system (Lafayette Instrument Company, IN, USA). Upon completion of the massage protocol, participants sat quietly in the lab until the 1 h postexercise sampling time-point.

### 2.4. Control Protocol

Immediately following postexercise blood sampling, performance, and soreness measurements, participants sat quietly in the laboratory for the 30 min period equivalent to the massage protocol and until the 1 h postexercise sampling time-point.

### 2.5. Ratings of Perceived Skeletal Muscle Soreness

Ratings of perceived skeletal muscle soreness were collected at pre-exercise; postexercise; and 1, 2, and 24 h postexercise on a 10-point Likert scale ranging from 1 “No soreness” to 10 “Agonizing”. These ratings were collected prior to commencing the tests of muscle function (jump tests).

### 2.6. Blood Sampling

Two 4.0 mL vacutainer tubes treated with 7.2 mg of EDTA (BD™ Vacutainer, USA) were collected from the antecubital vein while participants lay in the supine position on an examination table. Blood samples were collected prior to the tests of muscle function (jump tests) at pre-exercise; postexercise; and 1, 2, and 24 h postexercise time-points. Pre-exercise samples were taken at a minimum of 10 min prior to commencing the exercise protocol to allow the puncture site to clot. Postexercise samples were collected immediately following the exercise cool-down protocol, and the subsequent samples were collected 1, 2, and 24 h from the time the pre-exercise sample was collected. Vacutainer tubes were spun in a centrifuge (Universal 320 R, Hettich, Germany) for 15 min at 4 °C at 1560× *g*, and the plasma was aliquoted into 0.5 mL Eppendorf tubes and frozen at −80 °C until the time of assaying.

### 2.7. Muscle Function

Muscle function was measured using squat jump and drop jump heights measured using the Optojump™ Vertical Jump system (Microgate, USA). The Optojump™ uses an infrared beam to conduct a jump height computation calculated by flight time and body mass as independent variables. Jump tests were conducted pre-exercise; postexercise; and at 1, 2, and 24 h postexercise. Force generation indicated by jump height gives a reliable indication of integrated voluntary neuromuscular function. The squat jump test was performed with the participants’ hands on their hips in a standing position in the infrared beam of the Optojump™. They were then instructed to assume a squatted position with knees at 90°, pause for 1 s, and then jump maximally. This test allows only a concentric phase of force generation to be implemented, eliminating any contribution of prestretch activation of neuromuscular structures and passive elastic forces from connective tissue for force generation. As such, the squat jump test gives an indirect measure of muscle fiber activation, excitability, and functionality of contractile elements. Participants completed three attempts of the squat jump test at each time-point, and the average of the highest two jumps was used for statistical analyses to eliminate the effects of mis-jumps or submaximal efforts.

The drop jump test was used to measure the maximal voluntary force generated in a stretch-shortening cycle with the eccentric phase emphasized. This test includes a stretch-shortening cycle and therefore includes prestretch activation, stretch-reflex activation, and passive elastic contributions to force production [3]. The drop jump test was conducted from a 40 cm high box. Participants were instructed to start on the box with their hands on their hips. They were then verbally instructed to step off the box with their dominant foot, land in the infrared beam of the Optojump™, and immediately perform a maximal vertical jump. Participants completed three attempts of the drop jump test at each time-point, and the average of the highest two jumps was used for statistical analyses to eliminate the effects of mis-jumps or submaximal efforts.

These jump tests were used as tests of muscle function that are applicable to major movement types across athletic endeavors, as massage is widely used across many different sport types. Participants were familiarized with these two jump protocols prior to commencing any experimental procedures. Standardized instructions were given to each participant, with standardized technique correction if needed. Participants were reminded between jump attempts to give maximal effort.

### 2.8. Inflammatory Assays

Blood samples were analyzed for total plasma concentration of inflammatory signaling biochemicals using electrochemiluminescence-based solid-phase sandwich immunoassays (Meso-Scale Discovery, Rockville, MD, USA). A 30-plex multiarray assay for interferon (IFN), vascular endothelial growth factor (VEGF), interleukin-12 subunit p40 (IL-12p40), interleukin 12 subunit p70 (IL-12p70), interleukin-1 beta (IL-1β), interleukin-2 (IL-2), interleukin-4 (IL-4), interleukin-5 (IL-5), interleukin-6 (IL-6), interleukin-7 (IL-7), interleukin-8 (IL-8), interleukin-10 (IL-10), interleukin-13 (IL-13), interleukin-15 (IL-15), interleukin-16 (IL-16), eotaxin, eotaxin-3, interferon gamma-induced protein- 10 (IP-10), tumor necrosis factor alpha (TNFα), tumor necrosis factor beta (TNFβ), granulocyte-macrophage colony stimulating factor (GM-CSF), monocyte chemoattractant protein-1 (MCP-1), monocyte chemoattractant protein-4 (MCP-4), macrophage derived chemokine (MDC), macrophage inflammatory protein 1 alpha (MIP-1α), macrophage inflammatory protein 1 beta (MIP-1β), and thymus and activation regulated chemokine (TARC) was conducted according to the manufacturer’s instructions by an experienced laboratory technician. Samples and standards were run in duplicate. Interassay coefficient of variance (CV) values ranged from 4.9% to 15.2%, with CV < 20% deemed acceptable for statistical analysis.

### 2.9. Statistical Analyses

Data presented in text and figures represent the mean ± SD with a 95% confidence interval (unless otherwise indicated). Note that statistical analyses have not been corrected for multiple comparisons. Plasma concentration of inflammatory markers is expressed as change from baseline to account for interindividual variation and is expressed in pg/mL. Performance data are presented as the average of the highest two jump attempts of three total attempts expressed in cm to account for mis-jumps. Ratings of perceived soreness are expressed in points on a 10-point Likert scale. A two-way (five time-points × two recovery conditions) repeated measures analysis of variance (ANOVA) was used to determine the main effects for time and recovery condition and the interaction effects of time by recovery condition for all variables using SPSS™ version 24 (IBM, Armonk, NY, USA). The assumption of sphericity was tested by Mauchley’s W. In the case where the assumption of sphericity was violated, a Greenhouse–Geisser correction was used. All significant main effects were further analyzed with Tukey’s honestly significant difference (HSD) using GraphPad™ Prism software.

AUC for inflammatory concentration change over time provides a singular variable to indicate the overall inflammatory response to the exercise protocol. AUC was calculated only for inflammatory factors with significant time effects using the following methodology: the inflammatory factor concentration for each participant at postexercise and 1, 2, and 24 h postexercise was normalized to pre-exercise values. AUC was calculated using the trapezoidal method [20] in MATLAB R2017b (MATLAB, Natick, MA, USA). Paired *t* tests were used to identify significant differences in AUC as a gross measure of postexercise inflammation between experimental conditions. Correlations between AUC values for inflammatory markers with significant time effects and performance and soreness values at 24 h for all participants with complete datasets were conducted using Pearson’s product correlations on SPSS version 24 (IBM, Armonk, NY, USA).

## 3. Results

A total of nine male participants (mean age 23 ± 2 years) completed the exercise protocol on two different trial days. No significant differences were observed on measures of exertion, including time to complete sprints or RPE, and there were no differences in plasma cytokine concentrations at the pre-exercise time-point between experimental conditions. Data for one participant were excluded from the IL-10 statistical analyses due to CV exceeding 20%, and two participants for IL-6 (Table 2).

### 3.1. Inflammatory Markers

Significant main effects for time were observed for inflammatory markers, including IL-8 (*F*_(4,32)_ = 6.166, *p* < 0.001), IL-10 (*F*_(4,28)_ = 5.915, *p* < 0.001), IL-6 (*F*_(4,24)_ = 6.407, *p* < 0.001), TNFα (*F*_(4,32)_ = 4.756, *p* < 0.01), and MCP-1 (*F*_(4,32)_ = 16.082, *p* < 0.001) (Table 2). No significant changes were observed for other plasma analytes and thus are excluded from Table 2.

Plasma IL-8 was elevated from baseline (pre-exercise) at postexercise and 1 and 2 h postexercise in both massage and control experimental conditions (*p* < 0.05). IL-8 plasma concentration returned to a nonsignificant difference from baseline in the massage condition at 2 and 24 h postexercise, but in the control condition, IL-8 remained significantly higher than baseline at 2 h (*p* < 0.05) and 24 h (*p* < 0.04) postexercise. Plasma IL-10 was also elevated from baseline at 1 h postexercise in both massage and control experimental conditions (*p* < 0.02). IL-10 plasma concentration returned to nonsignificant change from baseline in both conditions at 2 and 24 h postexercise. Plasma IL-6 was elevated from baseline at postexercise as well as 1 and 2 h postexercise in both massage and control experimental conditions (*p* < 0.05). IL-6 plasma concentration returned to nonsignificant change from baseline in the control condition 24 h postexercise but remained elevated in the massage condition (*p* < 0.05).

Plasma TNFα was elevated from baseline at postexercise as well as 1 and 2 h postexercise in both massage and control experimental conditions (*p* < 0.05). TNFα plasma concentration returned to nonsignificant change from baseline in the massage condition at 24 h postexercise, while it remained significantly elevated from baseline in the control condition (*p* < 0.05). Plasma MCP-1 was elevated from baseline at postexercise and 1 h postexercise in both massage and control experimental conditions (*p* < 0.05). MCP-1 plasma concentration returned to nonsignificant change from baseline in the massage condition at 2 h postexercise, while it remained significantly elevated versus baseline in the control condition (*p* < 0.05). MCP-1 plasma concentration returned to nonsignificant concentrations in both conditions at 24 h postexercise.

No significant main effects for group or interaction effects were found for any of the inflammatory markers tested.

### 3.2. Performance and Soreness

Significant main effects for time were observed for performance and soreness measures (Table 3). A significant main effect for time was observed for squat jump height (*F*_(4,32)_ = 12.748, *p* < 0.01) and drop jump height (*F*_(4,32)_ = 15.330, *p* < 0.001). Squat jump height and drop jump height were significantly reduced compared with pre-exercise at all postexercise time-points in both massage and control conditions (*p* < 0.05). No significant main effects for group or interaction effects were observed for muscle performance measures.

A significant main effect for time was observed for ratings of perceived soreness (*F*_(4,32)_ = 13.266, *p* < 0.001). Compared with pre-exercise, perceived soreness was significantly greater at all postexercise time-points in both conditions (*p* < 0.05). No significant main effects for group or interaction effects were observed for muscle soreness ratings.

### 3.3. Correlation of Inflammatory Factors and Performance and Soreness

No differences were observed between massage and control experimental conditions for mean AUC for any of the inflammatory factors. Significant negative correlations were observed for MCP-1 AUC and drop jump height at 24 h (*r* = −0.469, *p* < 0.05) and squat jump height at 24 h (*r* = −0.510, *p* < 0.05). A significant positive correlation was observed between IL-10 AUC and drop jump height at 24 h (*r* = 0.563, *p* < 0.05). No significant correlations between AUC for IL-6, IL-8, or TNFα and performance variables at 24 h were observed.

## 4. Discussion

Massage therapy is a common postexercise recovery modality aimed to reduce DOMS and performance decrements by attenuating the inflammatory response to exercise [8,11,12,21]. Although this mechanism has been suggested by several authors [8,11,12,21], inflammatory processes are not commonly evaluated in tandem with performance and soreness metrics. The present study is the first to examine the effects of postexercise massage therapy using effleurage and neurolymphatic activation techniques on postexercise inflammatory signaling and subsequent muscle performance and soreness resulting from an intense bout of exercise.

The main finding of this study is that in a small sample of young healthy males, massage therapy employed immediately following an intense bout of intermittent sprint exercise was more effective than a control condition at facilitating return of circulating inflammatory signaling factors to baseline. More specifically, we observed significant increases in IL-8, IL-10, IL-6, TNFα, and MCP-1 postexercise in both the massage and control conditions, confirming that the exercise protocol was effective at inducing an inflammatory response. The massage therapy condition was associated with faster return to nonsignificant elevations from baseline of plasma concentrations for IL-8, IL-10, TNFα, and MCP-1 compared with the control condition, while IL-6 remained elevated in the massage condition but not the control condition. Although there were no main effects for group or interaction effects observed, these findings confirm our primary hypothesis as they indicate that massage can speed the time-course of inflammatory signaling, which aligns with previous studies that have observed inflammatory modulation at the tissue level following massage therapy [11,12]. Our findings support the use of massage therapy as a means of expediting the resolution of inflammatory signaling postexercise as indicated by concentrations of inflammatory mediators measured from systemic circulation.

Several studies have investigated the impact of massage therapy to treat DOMS and performance decrements experienced in the hours and days following an intense bout of exercise; however, results are variable [10]. Contrary to our hypothesis, in the current study, massage therapy did not reduce measures of pain or soreness compared to the nonmassage control group. It is unclear why we failed to observe changes in muscle soreness; however, our results agree with some previous studies that have examined the impact of massage on soreness measures. For example, one systematic review [10] of studies investigating the effects of massage therapy found that only four of seven studies reported reduced measures of pain or soreness with massage [22,23,24,25]. Furthermore, we did not observe any significant effects of massage therapy on muscle performance, indicated by squat jump and drop jump heights. While these findings do not support our hypothesis, they are consistent with some previous studies. For example, in the same systematic review [10], only 2 out of the 10 studies that examined the impact of massage on muscle performance reported improvements [26,27]. In another study, Tiidus et al. [28] demonstrated no significant difference between the isometric and dynamic peak torques between massage and control legs up to 96 h postexercise [28]. Therefore, while results are somewhat conflicting between studies, it appears that massage therapy may not be useful in recovering muscle function in the short term.

In the current study, we also examined the association between the AUC of inflammatory markers and muscle function (in the massage vs. control experimental conditions). We found a negative association between the proinflammatory factor MCP-1 with both drop jump and squat jump height (i.e., muscle function) and a positive correlation between anti-inflammatory factor IL-10 and squat jump height in both the massage and control conditions. MCP-1, also known as chemokine ligand 2 (CCL2), is a monocyte chemoattractant that is involved in recruiting monocytes to sites of inflammation or tissue damage which is released from the muscle in response to exercise [29]. IL-10 is an anti-inflammatory cytokine thought to promote a shift from inflammatory monocyte and T helper cell type 1 (Th1) activity towards a T helper cell type 2 (Th2) regenerative phenotype that may assist in the resolution of tissue disruption and inflammation at a cellular level [6,30]. Therefore, in the current study, we found that a proinflammatory marker was associated with worse muscle performance, and an anti-inflammatory cytokine was associated with better muscle performance. These associations support the idea that postexercise inflammation disrupts skeletal muscle function; however, these associations did not differ by massage versus control conditions despite resolution of inflammatory signaling at earlier time-points in the massage condition.

One possible explanation for the lack of difference in the massage versus control conditions is that the sample size of the current study was too small to reach statistical significance. It is also possible that the mechanical manipulation of muscles to induce mechanotransduction of anti-inflammatory cellular signaling [12] and a shift in local inflammatory cell phenotype to support healing [11] is the primary means of promoting functional recovery following an intense bout of exercise. Thus, stimulation of lymphatic circulation and clearance of cytokines and biochemical stress signals may only change inflammatory signaling at a systemic level with minimal nociceptive effects at the level of the muscle fiber [2]. However, further research designed to answer this question would be needed to confirm this.

The massage therapy protocol used for this study was a combination of effleurage inducing manual manipulation of the skeletal muscle belly and neurolymphatic activation to stimulate lymphatic circulation. Stimulation of lymphatic circulation has been suggested to enhance clearance of cytokines, creatine kinase (CK), and other noxious substances associated with inflammation and thought to contribute to soreness and functional disruptions of muscle [8]. As we employed both modalities to induce the anti-inflammatory effects of massage therapy, it is not possible to distinguish the effects of cellular inflammatory signal generation from the effects of enhanced clearance by lymphatic stimulation. A future comparison of these massage techniques is warranted to differentiate these two potential anti-inflammatory affects.

While our results confirm our hypothesis that massage therapy immediately postexercise can facilitate the resolution of inflammatory processes, additional limitations of our study are noteworthy. We measured inflammation using systemic concentrations of known inflammatory signaling factors in peripheral plasma samples. While this is indicative of systemic signaling processes, it is not possible to identify their source (i.e., skeletal muscle vs. immune cell vs. hepatic tissue) or the local inflammatory or regeneration processes that may be occurring [31,32]. A small sample size was used in the present study, which may have limited statistical power for determining differences between conditions or associations. Further, the exact inflammatory pathways mediated by cytokines and myokines are not fully understood, nor are their physiological implications. Some appear to promote inflammation, some to reduce it, and some may do either depending on the circumstances. While inflammation is thought to underlie the performance decrements and soreness experience following intense exercise, our current results do not support this, and the long-term implications of blunting of inflammatory signaling is not well understood.

## 5. Conclusions

In our small study of nine healthy young male athletes, massage therapy that included both mechanical manipulation of muscle tissue and neurolymphatic activation promoted resolution of systemic inflammatory signaling following high-intensity intermittent exercise. While massage did not appear to improve performance or soreness measurements, future studies that include larger sample sizes (including females) should confirm this. As well, future studies should investigate modalities of massage therapy for inflammatory effects and subsequent performance and soreness benefits to provide evidence-based best practice to athletes and practitioners and to further elucidate the anti-inflammatory effects of massage therapy. Overall, massage therapy appears to be an effective treatment regimen that resolves some exercise-related inflammation in male athletes following high-intensity intermittent exercise. Combined with other data that suggest that massage reduces DOMS and perceived fatigue, massage might be an effective postexercise recovery tactic that athletes should consider.

## Figures and Tables

**Table 1:** Descriptive and fitness characteristics of participants.

Factor	All Participants (*N* = 9)
Male sex (*N*; %)	100%
Age (years)	23 ± 2
Height (cm)	180.3 ± 5.79
Weight (kg)	84.4 ± 6.41
Avg power from Wingate test (watts kg^−1^)	9.08 ± 2.21
VO_2peak_ (mL kg minute^−1^)	61.32 ± 7.4

Notes: All values displayed as mean ± SD unless otherwise indicated.

**Table 2:** Change from baseline of inflammatory variables.

Variable	Postexercise	1 h Postexercise	2 h Postexercise	24 h Postexercise
IL-8 Control (*N* = 9)	0.73 ± 0.37 *	0.80 ± 0.35 *	0.46 ± 0.29 *	0.37 ± 0.09 *
	*p* < 0.001	*p* = 0.02	*p* = 0.48	*p* = 0.05
IL-8 Massage (*N* = 9)	1.01 ± 0.29 *	1.05 ± 0.36 *	−0.40 ± 0.15	−0.17 ± 0.22
	*p* < 0.001	*p* < 0.001	*p* = 0.11	*p* = 0.34
IL-10 Control (*N* = 8)	0.19 ± 0.12	0.58 ± 0.22 *	0.23 ± 0.12	0.003 ± 0.009
	*p* = 0.14	*p* = 0.03	*p* = 0.09	*p* = 0.87
IL-10 Massage (*N* = 8)	0.15 ± 0.04	0.16 ± 0.03 *	0.003 ± 0.06	−0.01 ± 0.07
	*p* = 0.06	*p* = 0.05	*p* = 0.74	*p* = 0.95
IL-6 Control (*N* = 7)	1.36 ± 0.46 *	1.04 ± 0.38 *	0.56 ± 0.26 *	0.13 ± 0.04
	*p* < 0.001	*p* = 0.02	*p* = 0.04	*p* = 0.45
IL-6 Massage (*N* = 7)	0.99 ± 0.29 *	0.68 ± 0.26 *	0.38 ± 0.12 *	0.45 ± 0.32 *
	*p* = 0.01	*p* = 0.04	*p* = 0.05	*p* = 0.04
TNFα Control (*N* = 9)	0.29 ± 0.10 *	0.28 ± 0.10 *	0.21 ± 0.07 *	0.14 ± 0.09 *
	*p* = 0.04	*p* = 0.04	*p* = 0.05	*p* = 0.05
TNFα Massage (*N* = 9)	0.21 ± 0.06 *	0.17 ± 0.08 *	0.11 ± 0.13 *	0.06 ± 0.13
	*p* = 0.05	*p* = 0.05	*p* = 0.05	*p* = 0.43
MCP-1 Control (*N* = 9)	61.534 ± 14.99 *	35.48 ± 8.09 *	28.06 ± 9.75 *	9.85 ± 10.05
	*p* < 0.001	*p* = 0.009	*p* = 0.01	*p* = 0.26
MCP-1 Massage (*N* = 9)	44.078 ± 11.93 *	30.79 ± 8.15 *	−1.99 ± 7.86	1.81 ± 6.47
	*p* = 0.002	*p* = 0.01	*p* = 0.87	*p* = 0.86

Notes: Only inflammatory variables with significant time effects are shown. All values indicate change from pre-exercise (baseline) concentrations measured in plasma in pg/mL units. * Indicates a statistically significant difference from baseline values at a level of *p* < 0.05.

**Table 3:** Summary of performance variables.

Variable	Baseline (Pre-Exercise)	Postexercise	1 h Postexercise	2 h Postexercise	24 h Postexercise
Squat Jump Height (cm)					
Control	35.54 ± 2.20	31.95 ± 1.83 *	32.27 ± 2.32 *	33.15 ± 2.39 *	32.51 ± 2.14 *
(*N* = 9)		*p* = 0.04	*p* = 0.04	*p* = 0.05	*p* = 0.05
Massage	36.02 ± 2.00	33.19 ± 1.61 *	33.16 ± 1.71 *	33.21 ± 1.74 *	34.14 ± 1.33 *
(*N* = 9)		*p* = 0.05	*p* = 0.05	*p* = 0.05	*p* = 0.05
Drop Jump Height (cm)					
Control	40.81 ± 2.49	36.84 ± 2.09 *	36.18 ± 2.45 *	37.82 ± 2.61 *	37.66 ± 2.62 *
(*N* = 9)		*p* = 0.03	*p* = 0.03	*p* = 0.04	*p* = 0.04
Massage	40.11 ± 1.76	36.19 ± 2.17 *	36.44 ± 2.01 *	36.50 ± 1.80 *	36.92 ± 1.37 *
(*N* = 9)		*p* = 0.03	*p* = 0.05	*p* = 0.05	*p* = 0.05
Perceived Soreness (unitless)					
Control	1.0 ± 0.4	3.5 ± 0.6 *	3.0 ± 0.3 *	2.7 ± 0.3 *	4.0 ± 0.5 *
(*N* = 9)		*p* = 0.03	*p* = 0.03	*p* = 0.04	*p* = 0.01
Massage	1.0 ± 0.5	4.4 ± 0.8 *	3.1 ± 0.7 *	3.5 ± 1.2 *	3.0 ± 0.7 *
(*N* = 9)		*p* = 0.02	*p* = 0.03	*p* = 0.03	*p* = 0.03

Notes: * Indicates a statistically significant difference from baseline values at a level of *p* < 0.05.
